# Optimization of the Proportioning and Microscopic Mechanism Study of Cement Mortar Prepared with Copper Tailings as Fine Aggregate

**DOI:** 10.3390/ma18112569

**Published:** 2025-05-30

**Authors:** Haizhou Li, Lu Zhang, Jianping Liu, Daozhong Chu, Jiaolong Ren

**Affiliations:** 1 School of Resources and Environmental Engineering, Shandong University of Technology, Zibo 255000, China; lihaizhou@sdut.edu.cn; 2 Key Laboratory for Liquid-Solid Structural Evolution & Processing of Materials of Ministry of Education, Shandong University, Jinan 250061, China; zlu@sdut.edu.cn; 3 School of Civil Engineering and Geomatics, Shandong University of Technology, Zibo 255000, China; liujp@sdut.edu.cn

**Keywords:** recycled cement mortar, copper slag aggregate, sulfate attack, Box-Behnken design, microstructure

## Abstract

To address the low resource utilization of copper tailings and high environmental impact of conventional river sand, this study innovatively integrates Box–Behnken design (BBD) with fractal theory to systematically investigate the performance optimization mechanisms of cement mortar incorporating copper tailings sand. A three-factor interaction model was developed through BBD experimental design, considering water–cement ratio (0.38–0.48), replacement ratio (10–30%), and binder–sand ratio (0.3–0.4), to elucidate the macroscopic performance evolution under multiparameter coupling effects. Fractal dimension analysis was employed to quantitatively characterize microstructural evolution. Experimental results demonstrate that the optimal parameters (water–cement ratio: 0.43, replacement ratio: 20%, binder–sand ratio: 0.35) yield superior performance, with 28-day compressive/flexural strengths reaching 61.88/7.14 MPa (12.3%/9.8% enhancement over the control group), and sulfate attack resistance showing 0.74% mass loss after 30 cycles. Microstructural analysis reveals reduced fractal dimension (D = 2.31) in copper tailings-modified specimens, indicating improved pore structure homogeneity. The enhanced performance is attributed to synergistic effects of micro-aggregate filling and pozzolanic reaction-driven C-S-H gel densification. This research establishes a novel multiscale methodology overcoming the limitations of conventional single-factor analysis, providing theoretical and technical support for high-value utilization of industrial solid wastes in construction materials.

## 1. Introduction

The composition of cement-based materials includes components such as cementitious materials, aggregates, and additives [1]. Aggregates are sourced from natural sand and gravel, serving the purposes of structural support and pore refinement. Statistics indicate that the concrete industry requires 100–200 billion tons of aggregates annually [2], with this demand increasing each year. Excessive extraction is bound to deplete natural resources, placing a significant burden on both resources and the environment. In order to address the environmental impact of excessive extraction of natural aggregates, the International Energy Agency recommends the use of alternative materials. Seeking substitutes for natural aggregates is currently at the forefront of solutions to this issue [3].

The mining and refining of copper produces a large amount of copper tailings [4]. Copper tailings consist of sand particles smaller than 6 mm, which remain after the ore is crushed and processed. For every ton of target metal extracted, about 200 tons of copper tailings are produced. The volume of tailings far exceeds that of the target metal [5,6]. Globally, 500 to 1000 million tons of copper tailings are produced each year [7]. And by 2019, 225 billion tons of copper tailings had been accumulated [8]. The total amount of copper tailings in China is approximately 2.4 billion tons [9]. Therefore, copper tailings are a significant industrial solid waste. The utilization rate of copper tailings is less than 10%, making it an underutilized resource [10]. The vast reserves of copper tailings cause many environmental issues, such as land occupation, groundwater and soil contamination with heavy metals, dam failure risks, and dust [11,12,13]. Currently, studies show that copper tailings contain high levels of SiO_2_, CaO, Al_2_O_3_, and other chemical components, which, after pretreatment, can partially replace traditional high-quality calcium and silica mineral materials. The properties of copper tailings are similar to those of natural sand, and their use as aggregates does not affect the volume stability of the matrix [14]. The physical and chemical properties of copper tailings sand are stable, making it a non-toxic aggregate [15]. Using copper tailings sand as an aggregate in mortar not only eliminates disposal costs and reduces land occupation but also reduces natural destruction and energy consumption caused by the production of fine sand, thus achieving the rational utilization of solid waste resources. This has substantial economic benefits and far-reaching social impacts.

The use of copper tailings sand as fine aggregate plays a significant role in enhancing the strength and durability of the matrix. Researchers have successfully investigated the workability [16], mechanical properties [17,18], and durability [19] of the base material, laying a solid foundation for future studies. Regarding workability, copper tailings sand, due to its high porosity and water absorption capacity, results in a reduced slump value for concrete [20]. Additionally, the shape of copper tailings sand is rough and angular, with higher water absorption compared to natural sand. Literature generally supports the idea that the addition of copper tailings sand reduces the flowability of the mix. Replacing 100% of the fine aggregate with copper tailings sand leads to a 38% decrease in slump [21]. To ensure proper workability, the replacement ratio of fine aggregate is typically kept below 45% [22]. Some studies indicate that the decrease in slump is not necessarily correlated with the replacement ratio due to the larger particle size of the tailings used, which does not significantly increase the surface area of the fine aggregates and has a limited effect on water absorption [23]. In terms of mechanical properties, with a constant water-to-binder ratio, incorporating an appropriate amount of copper tailings sand as a fine aggregate replacement can maintain the compressive, tensile, and flexural strength of the matrix [24]. The optimal replacement amount is between 30% and 50%, with a replacement ratio exceeding 60% being detrimental to strength development [25]. For a water-to-cement ratio between 0.40 and 0.45, the best replacement ratio of copper tailings sand is 30% to 40% [26]. The addition of a moderate amount of copper tailings sand reduces the total porosity of the matrix, increases its density, and enhances corrosion resistance and water permeability [27,28]. This is attributed to the decreased porosity, which helps delay the onset of corrosion, as chemical solutions find it more difficult to penetrate and degrade the material internally [29]. In conclusion, copper tailings sand is a promising alternative to natural sand.

Sulfate attack is a critical issue affecting the durability of mortar matrices. Under the combined influence of diurnal temperature variations and sulfate attack, the destruction rate of the mortar increases. Current studies on the performance of copper tailings sand as fine aggregate in mortar are still insufficient. Based on this, this study selects three influencing factors: water–cement ratio, copper tailings replacement rate, and binder–sand ratio, and uses the Box–Behnken design method from response surface methodology to analyze the sulfate resistance of mortar containing copper tailings sand. The model response target values include 28-day compressive strength, 28-day flexural strength, and mass variation of the mortar after 30 cycles of wet–dry treatment in a 5% sodium sulfate solution under standard curing for 28 days. Based on the model, the influence of each factor on the response values is analyzed, and the gray relational analysis theory is used to optimize the mix ratio of the mortar matrix. Through morphological analysis of SEM images and fractal dimension calculations, both qualitative and quantitative methods are employed to explain the mechanism by which copper tailings sand improves the mechanical and durability properties of the mortar from a microscopic perspective. This study provides a theoretical basis for the application and development of copper tailings sand.

## 2. Materials and Methods

### 2.1. Raw Materials and Properties

#### 2.1.1. Cement

The cement used in this experiment is ordinary Portland P.O.42.5 cement, Shandong Shanshui Group (Jinan, China), with an apparent density of 3.73 g/cm^3^. The testing methods for the strength at different ages of the cement were conducted in accordance with GB/T 17671-2021 [30], while the setting time test was performed following GB/T 1346 [31]. The technical performance results are presented in Table 1. The chemical composition of the cement was analyzed using the WJGS-010 X-ray fluorescence spectrometer produced by Rigaku Corporation in Tokyo, Japan. The experimental results are shown in Table 2.

Table 1 displays that the 3-day compressive strength of cement is 25.2 MPa, which exceeds the minimum requirement of 17 MPa; the 3-day flexural strength is 5.7 MPa, surpassing the 4 MPa threshold; the 28-day compressive strength is 44.3 MPa, meeting the 42.5 MPa standard; and the 28-day flexural strength is 8.1 MPa, exceeding the 4 MPa requirement. These results comply with the specifications outlined in the Chinese standard GB/T 17671-2021 [30]. Moreover, the cement exhibits an initial setting time of 88 min and a final setting time of 184 min, aligning with the criteria set forth in GB175-2023 [32].

Table 2 presents the X-ray fluorescence (XRF) analysis results of the cement, indicating that the primary components of the cement as a binding material are CaO and SiO_2_. CaO serves as the main source of cement strength, while SiO_2_ reacts with CaO to form calcium silicate. The content of MgO is measured at 0.98%, which falls within the acceptable range of ≤5.0%. These findings align with the specifications outlined in the Chinese standard GB175-2023 [32].

#### 2.1.2. Aggregates

**Figure 1:**
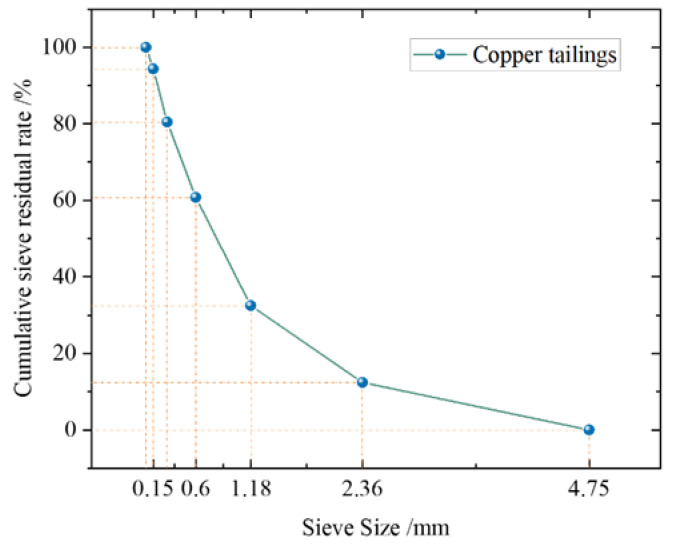
Grading of copper tailings.

The ISO standard sand produced by Xiamen Aisuo Standard Sand Co., Ltd., Fujian, China was selected as the control group fine aggregate for mortar. Copper tailings, a solid waste from the Yantai Copper Mine in Shandong, were characterized by a rough, angular, and porous surface. Sampling and testing procedures were conducted in accordance with the current industry standard JGJ52-2006 [33]. The copper tailings were used as a substitute for standard sand at equal masses. The fineness parameters of the aggregates significantly affect particle matching. Particle fineness analysis of the copper tailings was performed using the Dandong Baiti laser particle size analyzer. The cumulative particle size distribution curve is shown in Figure 1.

According to GB/T 25176-2010 [34], the gradation and classification of recycled fine aggregates are shown in Table 3. The copper tailings sand used in the test in this paper meets the gradation requirements of recycled fine aggregates within the gradation area of grade II.

According to the “Standard for Quality and Test Methods of Sand and Gravel for Ordinary Concrete” JGJ 52-2006 [33], the properties of standard sand and copper tailings sand are shown in Table 4.

The basic properties of the aggregates show that the apparent density of the copper tailings sand fine aggregate used in this study is 2360 kg/m^3^, which is lower than that of standard sand. According to GB/T 25176-2010 [34], “Recycled Fine Aggregates for Concrete and Standard Sand Mortar”, the requirement is that the apparent density should be greater than 2250 kg/m^3^, making it suitable for replacing fine aggregates. The water absorption rate of copper tailings sand is higher than that of standard sand. The mining, processing, and crushing of copper tailings sand generate numerous microcracks and pores, which contribute to its high water absorption capacity.

The composition of the copper tailings sand after grinding was analyzed using X-ray fluorescence (XRF), and the results are shown in Table 5.

Table 5 shows that the main components of the copper tailings sand aggregate are SiO_2_, FeO, CaO, and Al_2_O_3_. The copper tailings sand contains 4.7% CaO, which is a key factor influencing the pozzolanic effect of mortar materials. This indicates that the copper tailings sand powder has potential reactivity and can be used as a supplementary cementitious material. SiO_2_, FeO, and Al_2_O_3_ play significant roles in the formation of C-H-S gel during cement hydration. The combined content of these three components is 81.54%, suggesting that the copper tailings sand aggregate is primarily composed of silica-alumina (SiO_2_ and Al_2_O_3_) materials.

#### 2.1.3. Admixtures

Considering that copper tailings sand has more angular particles compared to standard sand, a water-reducing agent was added to the mortar mix at 0.5% by weight of cement. The water-reducing agent used in this experiment was produced by Henan Shuangjie Chemical Co., Ltd., Zhengzhou, China. The specifications of the water-reducing agent are shown in Table 6.

#### 2.1.4. Water

The mixing water used in the experiment was tap water from Zibo City, which complies with the requirements of the “Water for Concrete Mixing” standard (JGJ 63-2006) [35].

### 2.2. Experimental Design and Analytical Study

#### 2.2.1. The Influence of Replacement Ratio on the Workability

**Figure 2:**
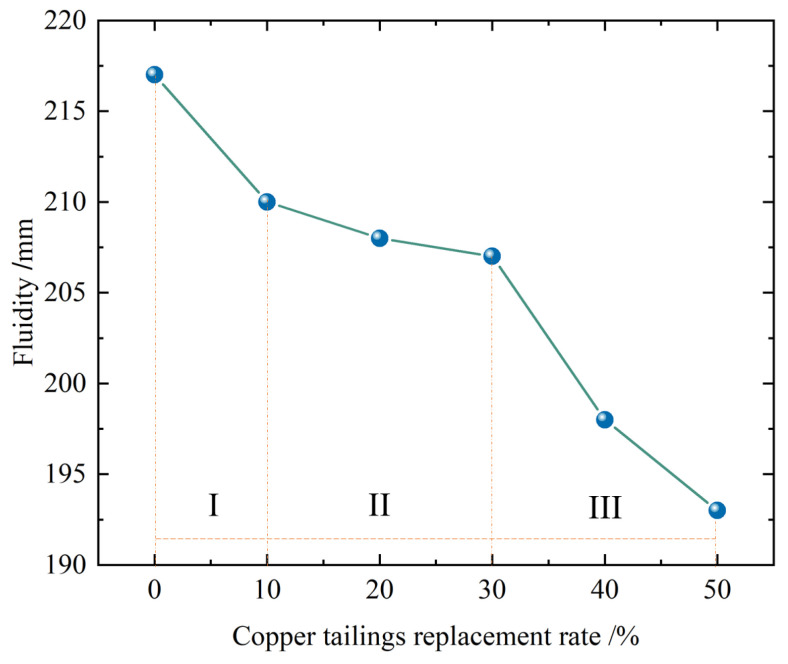
Influence of replacement rate of copper tailings on fluidity.

The results of the aggregate property tests in this study demonstrate that the water absorption of copper tailings is higher than that of standard sand. Using equal water content for both aggregates results in differences in the workability of the mixed mortar. Therefore, the impact of the copper tailings substitution rate on the workability of mortar cannot be overlooked. In this study, mortar mixes were prepared by replacing standard sand with copper tailings in the range of 0% to 50%. The effects of the replacement ratio on workability are illustrated in Figure 2.

As depicted in the figure, the slope of the lines represents the rate of change in workability. Based on the slope, the effect of copper tailings on the workability of mortar can be categorized into three stages.

In Stage I, with a copper tailings substitution rate of less than 10%, the workability decreases by 3.2%.

In Stage II, with a substitution rate of 10–30%, the workability decreases by 1.43%. The rate of decrease in the second stage is the slowest, indicating that the substitution rate of 10–30% has a minor impact on workability.

In Stage III, with a copper tailings substitution rate exceeding 30%, the workability decreases by 2.53%.

The experimental results on material properties reveal that copper tailings have a high water absorption rate and a sharper, irregular shape compared to the smooth, rounded standard sand, leading to higher frictional resistance and reduced flowability of the mortar. Taking all three stages into account, a copper tailings substitution rate of 10–30% is recommended.

#### 2.2.2. Impact of Binder–Sand Ratio on Strength

**Figure 3:**
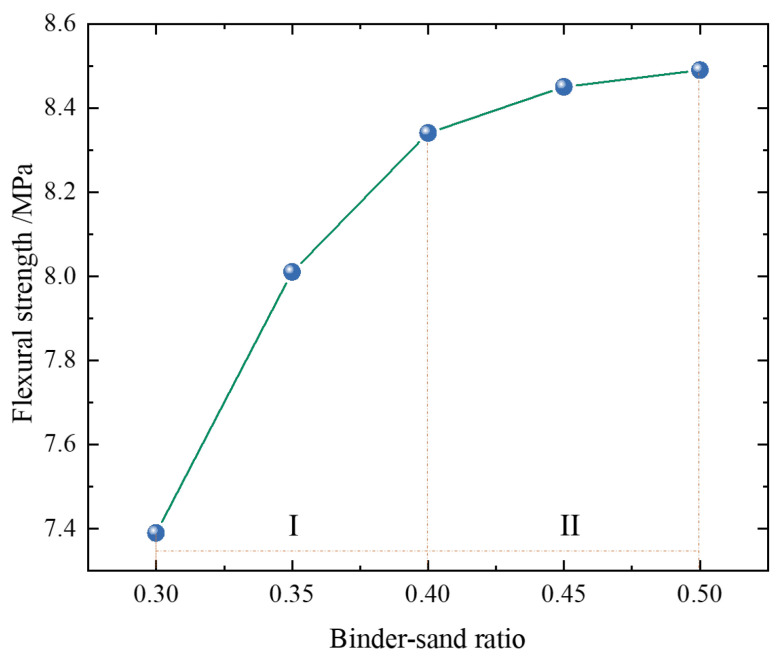
Influence of binder–sand ratio on flexural strength.

The binder–sand ratio refers to the mass ratio of the cementitious material to the aggregate in mortar. It is a crucial indicator influencing the mechanical properties of mortar. Additionally, as indicated in the literature [36], the binder–sand ratio has a significant impact on flexural strength. In this study, binder–sand ratios ranging from 0.3 to 0.5 were selected to assess the flexural strength of the mortar. The experimental results are presented in Figure 3.

As shown in Figure 3, with an increase in the sand-to-binder ratio, the flowability gradually increases. This trend can be divided into two stages.

Stage I: The increase is rapid, with flowability rising by 12.86%.

Stage II: The rate of increase slows down, with flowability increasing by only 0.47%, showing a less noticeable improvement.

As the amount of cementitious material increases, the ratio of the cement paste to the specific surface area of the aggregates also increases, which enhances the overall flowability of the material. Once the cement paste fully coats the aggregates, a threshold is reached, and further increases in cement content no longer result in significant improvements in strength. Considering economic factors, a sand-to-binder ratio of 0.3–0.4 is selected as the optimal range.

#### 2.2.3. Mix Design

1. Experimental design

The experimental design involves extracting representative samples within the design space to ensure a comprehensive reflection of the overall data characteristics, thereby achieving a certain level of representativeness. This enables the identification of patterns in the sample data with minimal experimentation. In this study, a Box–Behnken model with a three-factor, three-level, second-order response surface design was employed. The Box–Behnken design is particularly effective for experiments with a limited number of runs, offering high efficiency, minimal experimental iterations, and the ability to analyze the impact of each independent variable on the target response as well as their interactions. This design approach avoids generating unreasonable experimental data at extreme conditions [37].

The three influencing factors selected for the experiment are A: water–cement ratio, B: copper tailings replacement rate, and C: binder–sand ratio. Based on the experimental conclusions in Section 2.2.1 and Section 2.2.2, each factor is divided into three levels. The factor levels are shown in Table 7.

Based on the factor levels in Table 7, the mix design experiment consists of 17 groups. The curing age is set to 28 days, and the compressive strength, flexural strength, and corrosion mass change rate of the mortar after 30 cycles of wet–dry sulfate erosion are used as response variables for the model. The mix ratios and corresponding experimental results are shown in Table 8.

2. Response surface prediction model

The response surface experimental design uses an approximate polynomial to describe the functional relationship between the response variables and the factors. The form of the polynomial function needs to consider the selection of the degree. If the degree is too high, solving for the coefficients of the linear equations may lead to overfitting, causing significant deviations in the calculation results. Additionally, the design of interaction terms should be considered. Interaction terms primarily reflect the interactions between factors, and they should be retained in the experimental design when factors interact and influence each other. In this experiment, a quadratic polynomial expression was selected to describe the functional relationships among the three response variables, while retaining the interaction terms [38], as shown in Equation (1):

(1) $$Z=\omega_{0}+\sum_{i=1}^{n}\left(\omega_{i}m_{i}\right)+\sum_{i=1}^{n}\left(\omega_{ii}m_{i}^{2}\right)+\sum_{i=1}^{n}\sum_{j=1}^{n}\left(\omega_{ij}m_{i}m_{j}\right)+\psi$$

where *Z* is the predicted value of the mass loss response; $m_{i}$ represents the encoded values of the variables (factors); $m_{i}^{2}$ refers to the quadratic effects of the factors; $m_{i}m_{j}$ represents the interaction effects between the factors; $\omega_{0}$ is the constant regression coefficient; $\omega_{i}$ represents the regression coefficients for the linear effects of the factors; $\omega_{ii}$ refers to the regression coefficients for the quadratic effects of the factors; $\omega_{ij}$ refers to the regression coefficients for the interaction effects between the factors; ψ is the random error term; *n* is the number of variables (factors) involved.

3. Mix ratio optimization based on grey correlation

The grey correlation [39] analysis was introduced to analyze the 28-day compressive strength, 28-day flexural strength, and corrosion mass loss. The parameters were processed through Formula (2).

(2) $$k_{mn}=\frac{c_{mn}-n_{\min}c_{mn}}{n_{\max}c_{mn}-n_{\min}c_{mn}}\;(\mathrm{Target}\;\mathrm{maximum})\;k_{mn}=\frac{n_{\max}c_{mn}-c_{mn}}{n_{\max}c_{mn}-n_{\min}c_{mn}}\;(\mathrm{Target}\;\mathrm{minimum})$$

where $c_{mn}$ is the NTH performance index value of the m test, *m* = 1, 2, 3, …, 17 represents the number of tests; *n* = 1, 2, 3, indicates the number of target properties.

The grey relation number is calculated by Formula (3).

(3) $$\eta_{m}\left(n\right)=\frac{m_{\min}n_{\min}\left|k_{0}\left(n\right)-k_{m}\left(n\right)\right|+qm_{\max}n\max\left|k_{0}\left(n\right)-k_{m}\left(n\right)\right|}{\left|k_{0}\left(n\right)-k_{m}\left(n\right)\right|+qm_{\max}n\max\left|k_{0}\left(n\right)-k_{m}\left(n\right)\right|}$$

where $\eta_{m}$ is the grey correlation coefficient of the performance index; $k_{0}$ is the reference value, the reference value is generally selected 1; q is the recognition coefficient, the value range is 0–1, this paper takes 0.5.

The range value of each factor under each indicator is calculated through the following Formula (4), and then the sum of the range value of all indicators is calculated. The weight coefficient λ of the corresponding indicator can be obtained by dividing the range value of each indicator by the total range value, as shown in Formula (5).

(4) $$Z_{m,n}=\max\left\{S_{m,n,1},S_{m,n,2},\ldots ,S_{m,n,z}\right\}-\min\left\{S_{m,n,1},S_{m,n,2},\ldots ,S_{m,n,z}\right\}$$

(5) $$\lambda =\frac{\sum_{n=1}^{3}Z_{m,n}}{\sum_{m=1}^{17}\sum_{n=1}^{3}Z_{m,n}}$$

where *S* is the mean value of grey correlation coefficient at any level; Z is the mean range of grey correlation coefficients at any level.

Grey correlation degree Γ was calculated.

(6) $$\Gamma \left(k_{0}\right)=\sum_{n=1}^{3}\lambda \eta_{m}\left(n\right)$$

### 2.3. Experimental and Testing Methods

#### 2.3.1. Test Method

The workability was measured using the flowability test, following the “Method for Determining Flowability of Cement Mortar” (GB/T 2419-2005) [40]. The mechanical properties were tested using compressive strength after 28 days, with mortar specimens of 150 mm × 150 mm × 150 mm. Flexural strength specimens were 40 mm × 40 mm × 160 mm, and the tests were conducted according to the “Method for Testing Cement Mortar Strength (ISO Method)” (GB/T 17671-2021) [30]. The durability was evaluated using wet–dry cycling-sulfate erosion mass loss, with mortar specimens of 40 mm × 40 mm × 40 mm. The test was conducted according to the “Standard for Long-Term Performance and Durability Test Methods for Ordinary Concrete” (GB/T 50082-2024) [41]. The mass loss rate after a single wet–dry cycle of sulfate erosion was calculated using the following Formula (7):

(7) $$Z_{i}=\frac{Z_{e}-Z_{0}}{Z_{0}}$$

In the formula, Z*_i_* represents the average mass loss (%) for a set of specimens with the same mix ratio; Z*_e_* is the average mass after *e*e cycles of wet–dry cycling, in grams (g); and Z_0_ is the average initial mass of a set of specimens with the same mix ratio, in grams (g). According to the GB/T 50082-2024, if the mass loss rate exceeds 5%, the specimens are considered damaged or not meeting the requirements for actual engineering applications.

#### 2.3.2. SEM Testing

The SEM tests were conducted using a Quanta 250 scanning electron microscope, manufactured by FEI, Hillsboro, OR, USA. After 30 cycles of wet–dry cycling and sulfate erosion, the specimens were crushed into flat, angular-free, thin fragments. The fragments were then soaked in anhydrous ethanol to stop further hydration. Prior to testing, the fragmented specimens were removed, ensuring that the surface was clean and smooth, and were placed in an oven to dry at 40 °C for 24 h. After drying, the specimens were subjected to gold sputtering treatment for SEM observation.

## 3. Results and Analysis

### 3.1. Mix-Design Test Results

The test results of the mix proportion design are shown in Table 9.

### 3.2. Response Surface Analysis

#### 3.2.1. Establish the Response Surface Model

To perform regression fitting analysis for the 17 experimental data sets in Table 9, the objective values are determined by the factors A (water–cement ratio), B (copper tailings replacement rate), and C (binder–sand ratio). The regression fitting analysis is conducted based on the experimental data in Table 7 to establish quadratic polynomial regression equations for the compressive strength *C*_28_, flexural strength *F*_28_, and mass loss rate *Z*_28_ under dry–wet cycling sulfate erosion conditions. The resulting quadratic regression equations are shown in Table 10.

**Figure 4:**
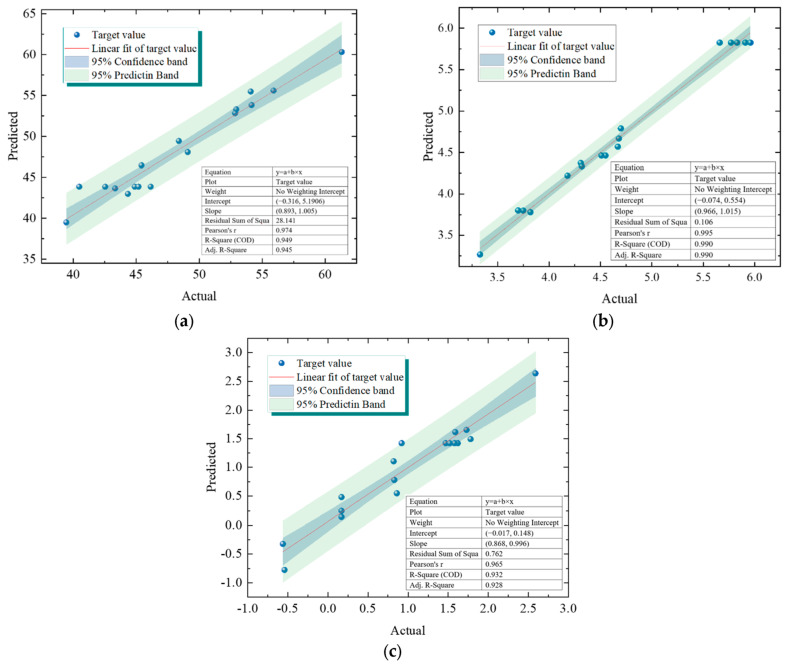
Actual and predicted values: (**a**) *C*_28_; (**b**) *F*_28_; (**c**) *Z*_28_.

To provide a more intuitive analysis of the model’s prediction accuracy, residual analysis was performed by comparing the actual and predicted values. Residuals contain important information regarding the underlying assumptions of the model and exhibit error characteristics. Analyzing the residuals can help assess the validity of the model assumptions and the reliability of the data. A high-quality regression model should have residuals that adhere to the characteristics of normality, randomness, homoscedasticity, and no autocorrelation. The residual analysis plots for the model are shown in Figure 4.

The residuals represent the difference between the model’s predicted values and the actual values. Residual analysis involves examining the validity of the model’s assumptions using the residuals and associated statistical metrics. Common types of residuals include raw residuals, internally studentized residuals, and externally studentized residuals. As shown in Figure 4, the predicted and actual values for *C*_28_, *F*_28_, and *Z*_28_ are distributed near the 45° line, approximating a linear distribution with good agreement. To further investigate the relationship between predicted and actual values, linear fitting was performed. The fitting accuracies for the *C*_28_, *F*_28_, and *Z*_28_ prediction models were 0.98, 0.99, and 0.96, respectively, indicating a significant linear relationship. Moreover, all data points fall within the 95% confidence interval and the 95% prediction band, which validates the applicability of the fitting results. This demonstrates that the response values predicted by the model have a high degree of reliability.

**Figure 5:**
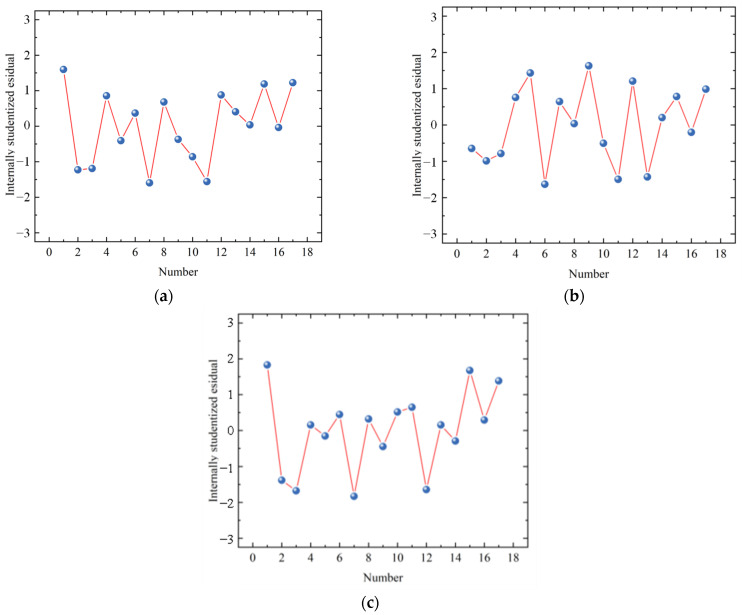
Internally studentized residuals: (**a**) *C*_28_; (**b**) *F*_28_; (**c**) *Z*_28_.

Traditional residual analysis assumes that the residuals across all data points have constant variance. However, the distribution of actual experimental data is complex and variable, leading to significant deviations from this assumption. To address the homoscedasticity assumption of standardized residuals, this study introduces internally studentized residuals (ISR), where the standard error is calculated individually for each data point to ensure the residuals are accurately and reliably represented. This approach is illustrated in Figure 5.

Figure 5 shows the absolute values of the ISR for the model data, with data points above 3 considered outliers. As seen in Figure 5a–c, all data points are accurate, with no outliers identified. The internally studentized residuals show no predictable or interpretable patterns with respect to the experimental sequence. The residuals are randomly distributed around the center line. Therefore, the relationship between the studentized residuals and the experimental sequence reflects the assumption of random distribution and homoscedasticity.

**Figure 6:**
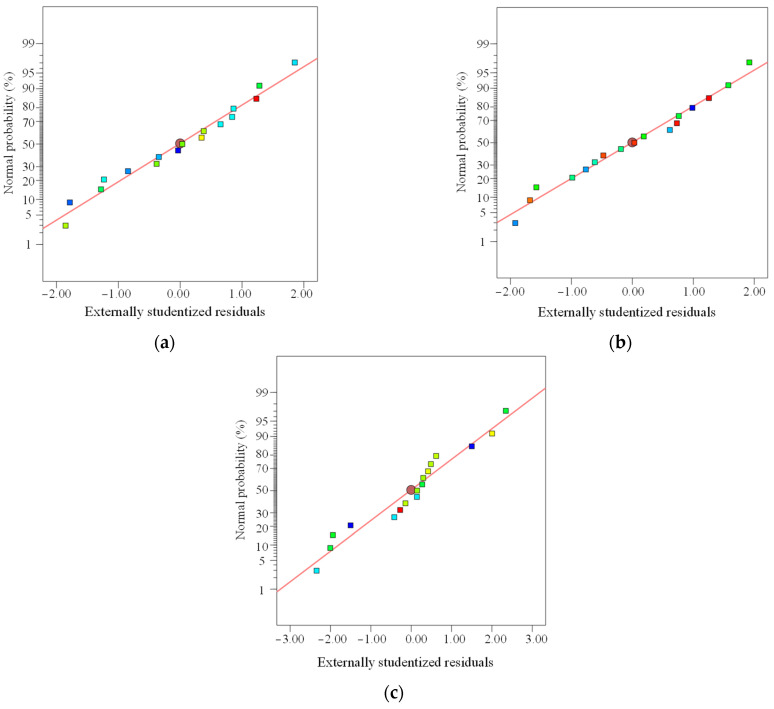
Externally studentized residuals: (**a**) *C*_28_; (**b**) *F*_28_; (**c**) *Z*_28_.

To ensure the accuracy of the residuals, the method of calculating the standard error for each data point while excluding the data point itself is referred to as externally studentized residuals. This approach is illustrated in Figure 6.

From Figure 6a–c, it can be observed that all data points are approximately linearly distributed, with points clustered near the diagonal line, indicating a good fit of the regression model equation and a significant relationship within the model. In the regression model data analysis, no phenomena of data deviation caused by measurement errors or incorrect records were observed, confirming that the response values predicted by the fitted model in this study are highly reliable. The established regression model can be used to predict actual experimental results.

#### 3.2.2. Model Significance Analysis

The significance of the regression model based on experimental results reflects the model’s applicability. In this study, analysis of variance (ANOVA) was conducted to test the significance of the regression model for the target values and to analyze the significance of the linear, quadratic, and interaction effects between the factors. The ANOVA results are presented in Table 11, Table 12 and Table 13. In the ANOVA of the target value regression model, the F-value represents the variance caused by changes in the corresponding factors. The model’s significance is determined by both the F-value and the *p*-value. A smaller *p*-value (≤0.05) and a larger F-value indicate a more significant model [42].

From the analysis of variance for the 28-day compressive strength regression model (Table 11), the *p*-value of the regression model is less than 0.0001, indicating that the model is highly significant. The *p*-value of the lack of fit term is 0.502, which is greater than 0.05, meaning the lack of fit is statistically insignificant compared to pure error. Therefore, the model exhibits good fitting accuracy across the entire design space. In the regression model equation’s linear terms, the influence of the three factors is ranked as C > A > B, with the binder-to-sand ratio having a significant effect on compressive strength. For the interaction terms, the influence on 28-day compressive strength follows the order of AB > AC > BC, with the AB interaction having a significant impact on the outcome. Among the quadratic terms, the effects of A^2^ and C^2^ are significant, with the order of influence being A^2^ > C^2^.

From the analysis of variance for the 28-day flexural strength regression model (Table 12), the F-value is 82.757, and the *p*-value is 0.00002, which is less than 0.0001, indicating that the model is highly significant. The *p*-value of the lack of fit term is 0.404, which is greater than 0.05, meaning the lack of fit is statistically insignificant compared to pure error. Among the factors, the water–cement ratio (A) has a significant effect, while the interaction effects of AB and BC are also significant. All three factors in the quadratic terms have significant effects.

From Table 13, it can be concluded that the F-value of the regression model is 10.823 and the *p*-value is 0.002, indicating that the effects of the selected factors on the sulfate resistance of the mortar specimens are significant. The *p*-values for factors B and C are both less than 0.05, indicating that the copper tailings sand replacement rate and binder-to-sand ratio have a more significant effect on the sulfate resistance of the mortar, with factor B having a greater influence than factor C. In the interaction terms, the *p*-value for the AB interaction is 0.004, and for BC, it is 0.01, confirming that the interactions of AB and BC have a significant impact on the experimental results. The quadratic terms A^2^ and B^2^ also significantly influence the mortar’s sulfate resistance. The *p*-value of the lack of fit term is 0.258, which is greater than 0.05, indicating that the lack of fit is statistically insignificant, suggesting high reliability of the experimental results, with no significant discrepancies due to experimental factors.

The reliability analysis of the regression model is shown in Table 14. The multiple correlation coefficient (R^2^) represents the degree of discrepancy between the predicted and actual response values, with values ranging from 0 to 1. The multiple correlation coefficient should be close to 1 to ensure a high degree of fit between the observed and predicted values. The difference between the adjusted R^2^ and the predicted R^2^ should be less than 0.2, ensuring good compatibility between experimental and predicted values, allowing the regression model to accurately describe the current experimental process. The adequacy precision reflects the model’s ability to resist disturbances, with values greater than 4 indicating that the model is reasonable.

The conclusions from Table 12 show that the multiple correlation coefficients (R^2^) for the C28, F28, and Z28 models are 0.964, 0.990, and 0.932, respectively. The differences between the adjusted R^2^ and the predicted R^2^ are 0.184, 0.058, and 0.03, all less than 0.2. The adequacy precision, which represents the ratio of effective signal to noise, is 15.934, 26.874, and 13.038, all greater than 4. The model exhibits high significance, and there is good compatibility between the predicted and experimental values, indicating that the model can be used for subsequent experimental analysis and optimization.

#### 3.2.3. Interaction Analysis of Significant Factors

**Figure 7:**
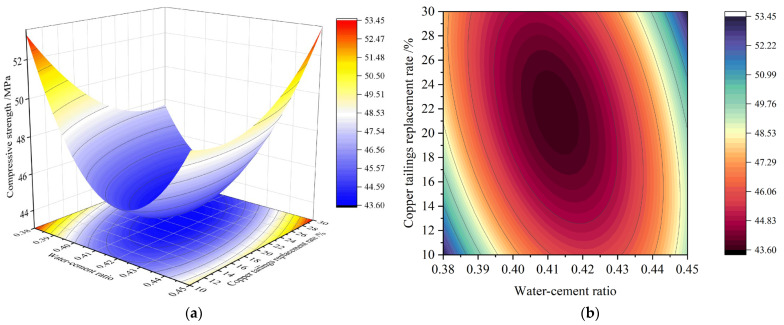
Response surface of 28 d compressive strength AB factor interaction: (**a**) 3D response surface diagram; (**b**) *contour map of AB factor*.

**Figure 8:**
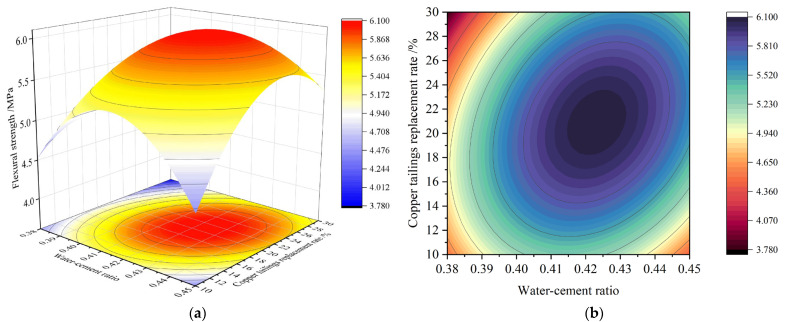

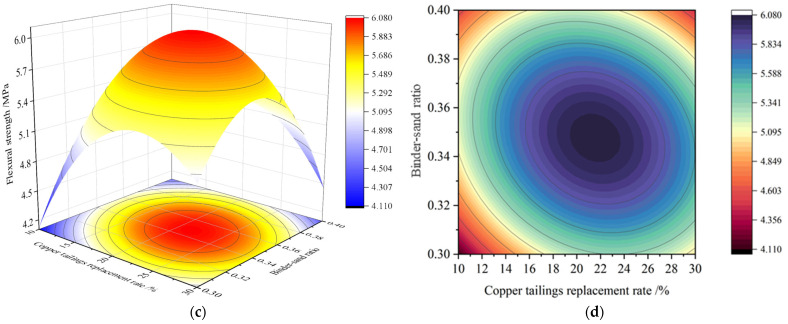
Response surface of flexural strength interaction for 28 d: (**a**) 3D response surface diagram of AB factor; (**b**) contour diagram of AB factor; (**c**) 3D response surface diagram of BC factor; (**d**) contour diagram of BC factor.

**Figure 9:**
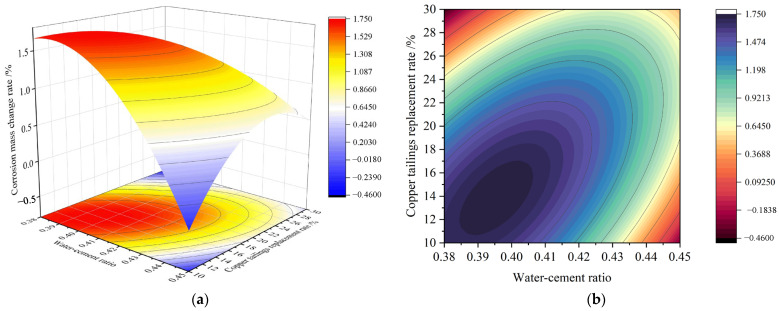

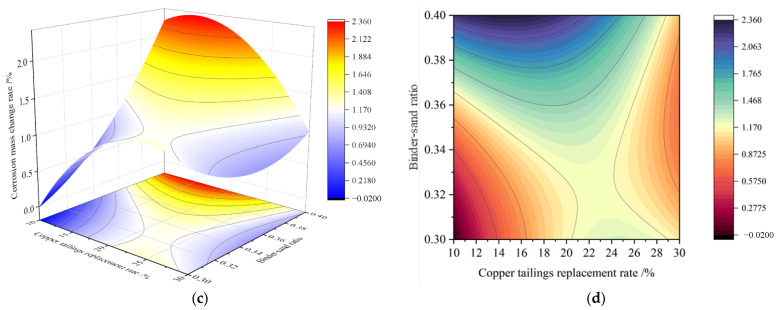
Interaction response surface of corrosion resistance: (**a**) 3D response surface diagram of AB factor; (**b**) contour diagram of AB factor; (**c**) 3D response surface diagram of BC factor; (**d**) contour diagram of BC factor.

The 3D response surface diagram of significant factors was used to analyze the influence trend of interaction of significant factors on response values, as shown in Figure 7, Figure 8 and Figure 9.

1. 3D response surface of compressive strength

Figure 7 presents the 3D response surface and corresponding contour plots illustrating the interaction between the water–cement ratio and copper tailings replacement rate on compressive strength.

In Figure 7, the shape of the contour lines (elliptical) indicates a significant interaction between these two factors, consistent with the results of the analysis of variance. The 3D response surface clearly shows that, with a fixed copper tailings replacement rate, the 28-day compressive strength initially decreases and then increases as the water–cement ratio rises. This phenomenon is caused by excess free water, which leads to the formation of voids during the hardening phase of the matrix. When the water–cement ratio is fixed, the effect of the copper tailings replacement rate on the 28-day compressive strength follows a trend of first decreasing and then increasing. The lower density and high crushing index of copper slag, compared to standard sand, result in lower strength, making it detrimental to matrix strength when replacing part of the standard sand. However, when added in appropriate proportions, the micro-powder effect from high crushing values helps to densify the matrix, enhancing its strength. The high water absorption property of copper slag is the key factor in the significant interaction of these two parameters.

2. 3D response surface of the flexural strength

Figure 8 illustrates the 3D response surface for the interaction between AB and BC on the 28-day flexural strength.

As shown in Figure 8, all three factors—A, B, and C—exhibit a trend where the 28-day flexural strength first increases and then decreases as their values increase. The elliptical contour lines on the response surface confirm the presence of an optimal value for both the AB and BC interactions within the selected range. The effect of C is seen in the increased binder content, which raises the ratio of the surface area of cement paste to aggregate, thereby enhancing the overall bond strength of the material and significantly improving flexural strength. Once the cement paste fully encapsulates the aggregate, further increases in cement content do not result in significant improvements in flexural strength.

3. 3D response surface of the corrosion resistance

Figure 9 shows that the elliptical contour lines on the response surface indicate a significant interaction between AB and BC on the sulfate resistance of the matrix.

In Figure 7, as the AB interaction increases, the corrosion resistance initially improves and then decreases. However, with an increase in factor C, the corrosion resistance continues to improve. The correlation between the water–cement ratio (A) and copper tailings replacement rate (B) is attributed to the higher water absorption of copper tailings sand compared to standard sand. This leads to less free water available for cement hydration under the same water–cement ratio, which somewhat affects the internal structure. The strong correlation between B (copper tailings replacement rate) and C (binder–sand ratio) is due to the higher specific surface area of copper tailings sand compared to standard sand, resulting in different amounts of binder required for equal masses of the two aggregates. Furthermore, the process of crushing copper tailings into aggregate produces a large amount of fine copper tailings powder. The inclusion of this powder accelerates the cement hydration process and improves the distribution of hydration products, making the mortar matrix more uniformly graded. The physical filling effect of the fine particles and the refinement of hydration products help block the penetration channels of sulfate ions, thereby improving the sulfate resistance of the mortar matrix.

### 3.3. Mix Ratio Optimization

The mix ratio optimization of fine aggregate mortar of copper tail sand takes three factors as dependent variables: water–cement ratio, copper tail sand replacement rate and glue sand ratio, and takes maximum compressive strength of 28 d, maximum bending strength of 28 d and minimum mass change rate after corrosion as targets. The target design is shown in Table 15.

The optimization results are shown in Table 16.

The optimal mix design parameters are as follows: water–cement ratio of 0.45, copper tailings sand replacement rate of 28.67%, and binder-to-sand ratio of 0.37. Mortar was prepared using the optimal mix design, and the results of the 30 dry–wet cycle sulfate attack mass loss test were compared with the predicted values from the model. The verification results are shown in Table 17.

As can be seen from Table 17, the error between the predicted value and the real value of the three target values is small, which is 0.42%, 5.62%, and 2.22%, respectively. The reliability of using a regression model to predict and optimize target parameters is high.

## 4. Microscopic Mechanisms

Cement-based material is a porous, multi-phase brittle composite material, and its microscopic properties are closely related to its macroscopic resistance to sulfate attack. Studying the microscopic characteristics of fine aggregate mortar of copper tail sand is helpful to reveal the mechanism of copper tail sand improving the corrosion resistance durability of mortar.

### 4.1. Microscopic Morphology Analysis

**Figure 10:**
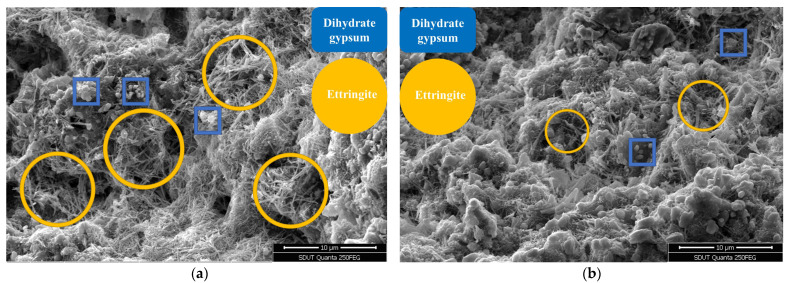
Typical corrosion morphology of standard mortar (*F*_1_): (**a**) *Enlarge the image 3000 times*; (**b**) *enlarge the image 5000 times*.

**Figure 11:**
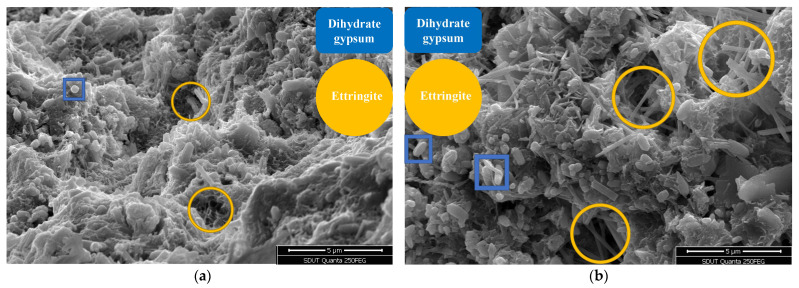
Typical corrosion morphology of fine aggregate mortar of copper tailings (*F*_2_): (**a**) *Enlarge the image 3000 times*; (**b**) *enlarge the image 5000 times*.

The microstructure of the mortar matrix will change under the attack of the ionic substance of the Na_2_SO_4_ solution. The mix ratio of water–cement ratio 0.45 and colloid-to-sand ratio 0.37 was selected. *F*_1_ used 100% standard sand, and *F*_2_ selected 28.67% copper-tail sand. The typical SEM images of the corrosion morphology of the two groups of substrates under different multiples under the condition of 30 dry–wet cycles and sulfate penetration are shown in Figure 10 and Figure 11.

During the hydration process of cement, the main hydration products are calcium-silicate-hydrate (C-S-H) and calcium hydroxide (CH). When external sulfate ions penetrate into the concrete, a series of chemical reactions occur. The corrosion process of mortar by Na_2_SO_4_ begins with the majority of sulfate ions reacting with CH to form gypsum, as shown in the reaction equation:

$$\mathrm{Ca}{\left(\mathrm{OH}\right)}_{2}{+\mathrm{SO}}_{4}^{2-}{+2H}_{2}{O=\mathrm{CaSO}}_{4}\cdot {2H}_{2}{O+2\mathrm{OH}}^{-}$$

In Figure 10 and Figure 11, the small flaky particles are gypsum dihydrate, which is brittle and detrimental to strength. As the reaction progresses, the reaction between CaSO_4_ and hydrated calcium aluminate leads to the formation of ettringite (3CaO·Al_2_O_3_·3CaSO_4_·31H_2_O, abbreviated as AFt), as expressed by the reaction equation:

$$\begin{array}{c} 3\mathrm{CaO}\cdot {\mathrm{Al}}_{2}O_{3}\cdot {6H}_{2}{O+3\mathrm{SO}}_{4}^{2-}+3\mathrm{Ca}{\left(\mathrm{OH}\right)}_{2}{+25H}_{2}O\\ =3\mathrm{CaO}\cdot {\mathrm{Al}}_{2}O_{3}\cdot {3\mathrm{CaSO}}_{4}\cdot {31H}_{2}{O+6\mathrm{OH}}^{-} \end{array}$$

In Figure 10 and Figure 11, the short, thick, and long needle-like substances are ettringite (AFt). Ettringite is a salt mineral with extremely low solubility and rich in crystalline water. The volume of ettringite is approximately 2.5 times that of the original hydrated calcium aluminate material. The formation of ettringite results in a significant increase in solid volume, forming radial growth in all directions, leading to high compressive stresses. The formation of ettringite causes internal expansion and damage to the mortar. The large distributed areas in Figure 10 and Figure 11 represent calcium-silicate-hydrate gel (C-S-H), which is a hydration product of cementitious materials and a primary source of strength and resistance to sulfate erosion [43]. The corrosion initiates with CH providing the necessary calcium ions for reactions. As the immersion time increases, the excess calcium hydroxide stored in the C-S-H gel is depleted, leading to the dissolution of C-S-H to continuously replenish calcium ions, resulting in the formation of numerous voids and cracks, consequently reducing the matrix density.

### 4.2. SEM Fractal Characteristics

**Figure 12:**
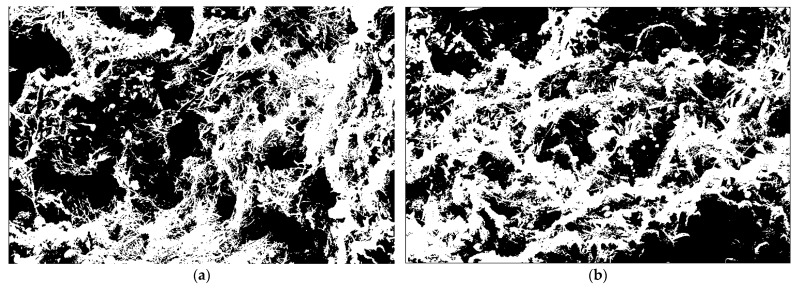
SEM image after binarization: (**a**) *F*_1_; (**b**) *F*_2_.

**Figure 13:**
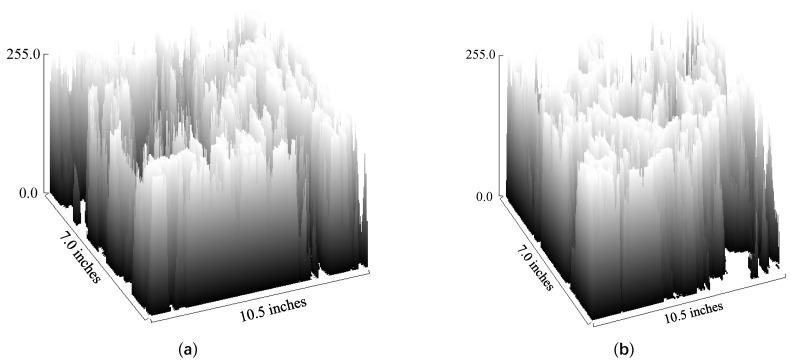
Gray distribution of SEM images: (**a**) *F*_1_; (**b**) *F*_2_.

There is a difference in density between the standard mortar and the optimal mix of copper tailings sand-based mortar. The typical SEM images in Figure 10a and Figure 11a show that the fracture surface of the mortar is highly uneven, with well-developed porosity. It is difficult to quantitatively describe the characteristics of the mortar fracture surface through direct observation of the images. The fractal dimension is an indicator used to measure the roughness of the image texture, allowing the quantitative description of visual differences in the image. In this study, the box-counting method was used for quantitative analysis of the images, and the Otsu method was employed to determine the binarization threshold [44]. After binarizing the SEM images into black-and-white images, the images were segmented into non-overlapping target regions, as shown in Figure 12. The grayscale values ranging from 0 to 255 were assigned to the Z-axis, and the length and width of the image were taken as the X and Y axes, respectively. The three-dimensional grayscale distribution is shown in Figure 13. The alternating black and white areas represent the roughness variations of the SEM image. In the image, the white areas represent the mortar matrix, while the black areas represent pores or cracks.

As calculated from Figure 13, the grayscale difference for the standard mortar in Figure 13a is 1935, while the grayscale difference for the optimal mix of copper tailings sand-based mortar in Figure 13b is 1669. The large pixel differences in the standard mortar indicate higher roughness and lower density compared to the copper tailings sand-based mortar. A box of side length h is used, and the number of black areas is denoted as *N*(h), $h=1,\;2,\;4,\;\ldots ,\;2^{i}(i=0,\;1,\;2,\;3,\;\ldots )$. The image is divided into regions with side lengths of 1, 2, 4,..., 2^i^ pixels, resulting in the box counts *N*(1), *N*(2), *N*(3), and *N*(4) [44]. As *k* approaches 0, the fractal dimension is determined using the box-counting method, as shown in Equation (8).

(8) $$D=\underset{h\to 0}{\lim}\frac{\mathrm{lg}N\left(h\right)}{\mathrm{lg}h}$$

From the above formula, the smaller h is, the more accurate the fractal dimension is, and the minimum h value is a single pixel point. Formula (9) is obtained by fitting *N*(h) corresponding to different h using the least squares method [45]:

(9) $$\mathrm{lg}N\left(h\right)=D\cdot \mathrm{lg}h+b$$

**Figure 14:**
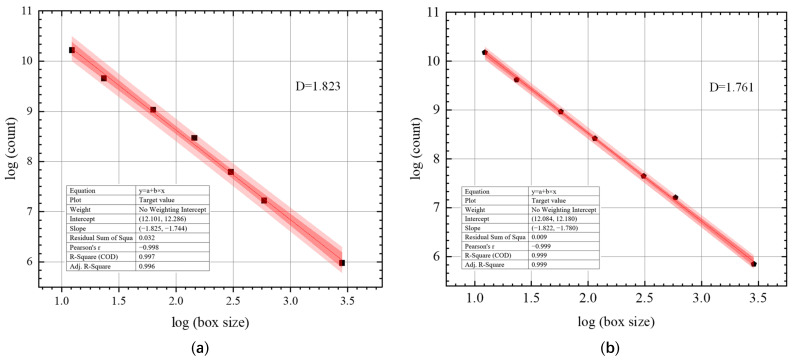
Fractal dimension and fitting accuracy: (**a**) *F*_1_; (**b**) *F*_2_.

The slope k of the line drawn according to the equation is the desired fractal dimension. The slope is negative, and the fractal dimension is D = −k. The calculation of fractal dimension and fitting accuracy are shown in Figure 14.

As shown in Figure 14, the fractal dimension of the mortar cross-sectional pores is calculated using the box-counting method, with correlation coefficients greater than 0.99, indicating that the SEM images of the mortar cross-section exhibit fractal characteristics. The fractal dimension can serve as a characteristic parameter to describe the roughness of the mortar cross-section. Under the same magnification, the roughness of the matrix increases with the fractal dimension. The SEM cross-sectional fractal dimension of copper tailings sand-based mortar is 1.761, lower than the 1.823 of the standard mortar, suggesting that the inclusion of copper tailings sand reduces the initial damage to the mortar’s internal cross-section. This conclusion is consistent with the findings in the literature [46]. Due to the high water absorption, porous structure, and rough, irregular surface of copper tailings sand, it can tightly embed into the cement matrix, improving the initial density. The copper tailings powder adhering to the surface of the copper tailings sand plays a physical filling role during the early stage of paste hardening, and the later volcanic ash effect increases the thickness of the interfacial transition zone. As a result, the copper tailings sand-based mortar exhibits stronger bonding at the microstructural level and an improved interfacial transition zone, leading to enhanced corrosion resistance.

## 5. Conclusions

In this study, the mix proportions of cement mortar incorporating copper tailings sand as fine aggregate were optimized using response surface methodology. The workability, mechanical properties, and durability of the mortar were systematically evaluated, and the influence of copper tailings sand on mortar performance was elucidated from a microstructural perspective. The main conclusions are as follows:

(1) The incorporation of copper tailings sand reduced the flowability of the mortar to some extent. However, maintaining the replacement rate within the range of 10–30% effectively balanced workability and economic efficiency.

(2) At a fixed 20% replacement level of standard sand with copper tailing sand, the binder–sand ratio had a positive effect on flowability. The increase in flowability exhibited two distinct stages: in Stage I, flowability increased by 12.86%; in Stage II, the growth rate slowed, with a further increase of 0.47%. An optimal binder-to-sand ratio was found within the range of 0.30–0.40.

(3) Response surface analysis indicated that the binder-sand ratio (A) and the interaction between factors water–cement ratio (A) and copper tailings replacement rate (B) had significant effects on 28-day compressive strength. The water-to-cement ratio, as well as the interactions of A–B and B–C, significantly influenced 28-day flexural strength. The replacement rate of copper tailing sand, the binder-to-sand ratio, and the interactions A–B and B–C also had notable effects on sulfate resistance.

(4) A multi-objective optimization was performed by fitting second-order polynomial regression models for the three target properties and applying grey relational analysis. The optimal parameter values were determined as a water-to-cement ratio of 0.45, copper tailings sand replacement rate of 28.67%, and binder-to-sand ratio of 0.37. The predicted values showed good agreement with experimental results, with relative errors of 0.42%, 5.62%, and 2.22%, respectively.

(5) Fractal analysis of SEM images after sulfate attack revealed that the fractal dimension of the fracture surface for the optimized copper tailing mortar was 1.761, lower than that of the standard mortar (1.823). This suggests that the inclusion of copper tailings sand reduces initial microstructural damage within the mortar. Overall, copper tailings sand improved the corrosion resistance and durability of the mortar.

## Figures and Tables

**Table 1:** Test results of technical properties of cement.

Strength Class	3 d Compressive Strength /MPa	3 d Bending Strength /MPa	28 d Compressive Strength /MPa	28 d Bending Strength /MPa	Apparent Density /(g/cm^3^)	Initial Setting Time /min	Final Setting Time /min
42.5	25.2	5.7	44.3	8.1	3.73	88	184

**Table 2:** Cement chemical composition analysis table.

**Ingredient**	CaO	SiO_2_	CO_2_	Al_2_O_3_	Fe_2_O_3_	MgO	K_2_O	Loss
**Content/%**	50.77	23.94	12.81	8.15	2.63	0.98	0.47	0.25

**Table 3:** Fine aggregate grading table.

**The Screen Hole Side Length/mm**		4.75	2.36	1.18	0.6	0.3	0.15
**Cumulative** **screening** **residue/%**	**Distribution zone I**	0–10	5–35	35–65	71–85	80–95	85–100
	**Distribution zone II**	0–10	0–25	10–50	41–70	70–92	80–100
	**Distribution zone III**	0–10	0–15	0–25	16–40	55–85	75–100
	**Copper-tailings sand gradation area**	0	12.4	32.5	60.8	80.5	94.4

**Table 4:** Basic properties of fine aggregate.

Aggregate Property	Apparent Density /(kg/m^3^)	Loose Density /(kg/m^3^)	Crushing Index /%	24 h Water Absorption /%
Standard sand	2520	1480	14.28	1.26
Copper tailings sand	2360	1052	17.66	2.42

**Table 5:** X-ray fluorescence spectrum analysis.

**Ingredient**	SiO_2_	CaO	FeO	Fe_3_O_4_	Al_2_O_3_	CuO	MgO	S	LOSS
**Content/%**	41.2	4.7	34.2	5.57	6.34	2.6	4.12	0.87	0.4

**Table 6:** Performance index of water-reducing agent.

Index	Water Reduction Rate/%	Gas Content/%	pH	Density/(g/cm^3^)
**Numerical value**	30	2.6	7.8	1.174

**Table 7:** Test factor level table.

Level	Influencing Factor		
	A	B/%	C
−1	0.38	10	0.30
0	0.43	20	0.35
1	0.45	30	0.40

**Table 8:** Mix design.

Group	A	B/%	C	Cement /(kg/m^3^)	Standard Sand /(kg/m^3^)	Copper Tailings Sand /(kg/m^3^)	Water /(kg/m^3^)	Water Reducing Agent /(kg/m^3^)
1	0.43	20	0.35	467	1111	222	201	2.3
2	0.45	20	0.3	415	1154	231	187	2.1
3	0.43	20	0.35	467	1111	222	201	2.3
4	0.43	20	0.35	467	1111	222	201	2.3
5	0.45	10	0.35	467	1212	121	210	2.3
6	0.43	30	0.4	514	989	297	221	2.6
7	0.45	30	0.35	467	1025	308	210	2.3
8	0.38	30	0.35	467	1025	308	177	2.3
9	0.38	20	0.3	415	1154	231	158	2.1
10	0.38	20	0.4	514	1072	214	195	2.6
11	0.38	10	0.35	467	1212	121	177	2.3
12	0.43	20	0.35	467	1111	222	201	2.3
13	0.43	10	0.4	514	1169	117	221	2.6
14	0.43	10	0.3	415	1259	126	178	2.1
15	0.43	30	0.3	415	1065	320	178	2.1
16	0.43	20	0.35	467	1111	222	201	2.3
17	0.45	20	0.4	514	1072	214	231	2.6

**Table 9:** Mix-design results.

Group	28 d Compressive Strength/MPa	28 d Bending Strength/MPa	Corrosion Mass Change Rate/%
1	52.94	4.55	1.59
2	48.39	4.31	−0.56
3	49.1	3.33	−0.54
4	54.16	4.70	0.17
5	45.43	3.75	0.82
6	43.33	4.67	0.17
7	55.91	3.70	1.73
8	61.31	4.51	1.78
9	44.34	4.18	0.86
10	39.46	4.32	0.83
11	52.85	4.68	2.59
12	54.1	3.82	0.17
13	44.89	5.83	1.52
14	45.19	5.96	0.92
15	45.16	5.91	1.47
16	42.54	5.77	1.58
17	41.48	5.66	1.62

**Table 10:** Target value prediction model.

Curing Period	Prediction Model of Mass Loss Rate
Compressive strength	*C*_28_ = 1117.479 − 4260.103A − 4.64B − 962.537C + 6.864AB + 1071.428AC + 3.065BC +4531.836A^2^ + 0.017B^2^ + 836.6C^2^
Flexural strength	*F*_28_ = −156.527 + 567.277A − 0.019B + 245.736C + 1.15AB − 15.714AC − 0.5BC − 692.244A^2^ − 0.007B^2^ − 328.2C^2^
mass loss rate	*Z*_28_ = −48.702 + 338.463A − 0.223B − 99.695C + 2.042AB + 100AC − 1.195BC − 507.959A^2^ − 0.006B^2^ + 130.1C^2^

**Table 11:** Variance analysis of 28 d compressive strength regression model.

Source	Sum of Squares	df	Mean Square	F-Value	*p*-Value	Significance
Model	555.342	9	61.704	21.290	0.0002	Significant
A	1.814	1	1.814	0.626	0.454	
B	0.361	1	0.361	0.124	0.734	
C	332.949	1	332.949	114.877	0.0001	Significant
AB	23.088	1	23.088	7.966	0.025	Significant
AC	14.062	1	14.062	4.852	0.063	
BC	9.394	1	9.394	3.241	0.114	
A^2^	129.764	1	129.764	44.772	0.0002	Significant
B^2^	12.806	1	12.806	4.418	0.073	
C^2^	18.418	1	18.418	6.354	0.039	Significant
Lack of Fit	8.361	3	2.787	0.934	0.502	not significant

**Table 12:** Variance analysis of 28 d regression model of flexural strength.

Source	Sum of Squares	df	Mean Square	F-Value	*p*-Value	Significance
Model	11.464	9	1.273	82.757	0.00002	Significant
A	1.022	1	1.022	66.426	0.0008	Significant
B	0.300	1	0.300	19.510	0.003	Significant
C	0.005	1	0.005	0.358	0.568	
AB	0.648	1	0.648	42.101	0.0003	Significant
AC	0.003	1	0.003	0.196	0.670	
BC	0.25	1	0.25	16.242	0.004	Significant
A^2^	3.027	1	3.027	196.711	0.00002	Significant
B^2^	2.403	1	2.403	156.137	0.00004	Significant
C^2^	2.834	1	2.834	184.159	0.00002	Significant
Lack of Fit	0.052	3	0.017	1.244	0.404	not significant

**Table 13:** Variance analysis of mass corrosion regression model.

Source	Sum of Squares	df	Mean Square	F-Value	*p*-Value	Significance
Model	11.367	9	1.263	10.822	0.002	Significant
A	0.520	1	0.520	4.457	0.072	
B	1.852	1	1.852	15.876	0.005	Significant
C	1.611	1	1.611	13.804	0.007	Significant
AB	2.044	1	2.044	17.522	0.004	Significant
AC	0.122	1	0.122	1.049	0.339	
BC	1.428	1	1.428	12.236	0.010	Significant
A^2^	1.630	1	1.630	13.969	0.007	Significant
B^2^	1.696	1	1.696	14.536	0.006	Significant
C^2^	0.445	1	0.445	3.816	0.091	
Lack of Fit	0.488	3	0.162	1.986	0.258	not significant

**Table 14:** Model reliability analysis table.

Model	R^2^	Adjusted R^2^	Predicted R^2^	Adeq Precision
*C*_28_	0.964	0.919	0.735	15.934
*F*_28_	0.990	0.978	0.920	26.874
*Z*_28_	0.932	0.846	0.816	13.038

**Table 15:** Target design of response surface.

Name	Goal	Lower Limit	Upper Limit
A	is in range	0.38	0.48
B	is in range	10	30
C	is in range	0.3	0.4
Compressive strength/MPa	maximize	39.46	61.31
Flexural strength/MPa	maximize	3.33	5.96
Corrosion mass change rate/%	minimize	−0.56	2.59

**Table 16:** Optimal mix ratio.

Group	A	B	C	Compressive Strength/MPa	Flexural Strength/MPa	Corrosion Mass Change Rate/%	Result
1	0.45	28.669	0.365	56.171	4.794	0.331	Selected
2	0.45	28.731	0.365	56.193	4.787	0.324	
3	0.45	28.694	0.365	56.286	4.784	0.332	

**Table 17:** Error between predicted value and true value of response surface optimization.

Index	Compressive Strength/MPa	Flexural Strength/MPa	Corrosion Mass Change Rate/%
Predicted value	62.14	6.76	−0.721
Actual value	−0.26	0.38	−0.016
Error (%)	0.42	5.62	2.22

## Data Availability

The original contributions presented in this study are included in the article. Further inquiries can be directed to the corresponding authors.
